# A Multifunctional Hydrogel Incorporating Luteolin-Encapsulated ROS-Responsive Nanoparticles and Stem Cells Promotes Bacterial-Infected Wound Healing

**DOI:** 10.3390/pharmaceutics18010098

**Published:** 2026-01-12

**Authors:** Jingjing Wang, Rui Ni, Ziwei Li, Jianhong Chen, Yao Liu

**Affiliations:** 1 Department of Pharmacy, Daping Hospital, Army Medical University, Chongqing 400042, China; wangjingjing@tmmu.edu.cn (J.W.); nirui719@tmmu.edu.cn (R.N.); liziwei@tmmu.edu.cn (Z.L.); 2 Central Research Laboratories, Chongqing General Hospital, Chongqing University & Chongqing Academy of Medical Sciences, Chongqing 401147, China

**Keywords:** multifunctional wound dressing, antibacterial, antioxidant, anti-inflammatory, wound healing

## Abstract

**Background/Objectives:** Wound healing represents a pervasive and urgent clinical challenge. Hard-to-heal chronic wounds are frequently complicated by infections, inflammatory responses, and oxidative stress. Currently, wound dressings are broadly categorized into dry and moist types, with moist wound dressings for chronic wounds accounting for approximately 70% of market revenue. Recently, adipose-derived stem cells (ADSCs), which possess self-renewal and multi-lineage differentiation capabilities, have emerged as a promising strategy for promoting tissue regeneration and wound repair. **Methods:** In this study, we developed a novel luteolin nanoparticle–ADSCs composite hydrogel (GelCA@LUT@ADSCs). This system was constructed by first encapsulating ADSCs within a chitosan/alginate hydrogel (GelCA), followed by coating the hydrogel with luteolin-loaded nanoparticles (LUT@NPs). **Results:** The sustained release of LUT@NPs from the hydrogel modulates the wound microenvironment, enhancing the pro-healing functions of ADSCs at the wound site. The GelCA hydrogel exhibited excellent biocompatibility. Both in vitro and in vivo results demonstrated that GelCA@LUT@ADSCs treatment effectively reduced inflammation, promoted angiogenesis and collagen deposition, stimulated cell proliferation and migration, and polarized macrophages toward an anti-inflammatory, pro-healing M_2_ phenotype, thereby accelerating wound healing. **Conclusions:** Overall, this innovative therapeutic approach provides a novel strategy for wound management through a synergistic division of labor between pharmaceutical agents and stem cells.

## 1. Introduction

In recent years, dysregulated processes such as infection, persistent inflammation, and oxidative stress have been recognized as major impediments to clinical wound healing [1,2]. While an appropriate inflammatory response initiates vasodilation, immune activation, and cytokine release to clear pathogens and stimulate angiogenesis, excessive or prolonged inflammation exacerbates tissue damage and impairs regeneration [3]. Concurrently, reactive oxygen species (ROS)—including superoxide ions and hydrogen peroxide (H_2_O_2_)—disrupt redox homeostasis, amplify inflammatory signaling, suppress collagen synthesis, and inhibit cell proliferation, thereby delaying wound closure [4,5]. Furthermore, infection risk remains a critical concern, particularly in immunocompromised individuals. Conventional antibiotics such as vancomycin carry potential adverse effects, including severe cutaneous reactions, underscoring the need for safer therapeutic alternatives [6].

Stem cells have garnered significant attention in regenerative medicine due to their self-renewal capacity and multilineage differentiation potential. In wound healing, stem cell-based strategies offer novel approaches for advanced dressing design. Adipose-derived stem cells (ADSCs), a type of mesenchymal stem cell, contribute to tissue repair through immunomodulation, angiogenesis, and paracrine signaling [7,8,9]. They secrete anti-inflammatory cytokines such as Interleukin 10 (IL-10) and Transforming growth factor-β (TGF-β) to suppress excessive inflammation, while releasing pro-regenerative factors including Vascular endothelial growth factor (VEGF) and TGF-β to enhance vascularization and matrix remodeling [10,11]. ADSCs further mitigate fibrosis by modulating TGF-β1-mediated fibroblast activation and promoting collagen turnover via matrix metalloproteinase regulation. Recent studies confirm elevated levels of VEGF and IL-6 in wound exudates following ADSC therapy, with animal models demonstrating improved healing through enhanced angiogenesis and regulated cytokine secretion [12].

Conventional wound dressings based on gauze and sponge suffer from limited exudate absorption, poor breathability, high allergenicity, and dependency on adjunct fixation. In response, emerging strategies in tissue engineering and nanomedicine leverage stem cells, growth factors, and scaffold materials to modulate gene expression, enhance cellular activities, and improve the wound microenvironment, thereby accelerating healing [13,14,15]. Among advanced dressings, hydrogels—formed via physical or electrostatic crosslinking into three-dimensional networks—serve as ideal stem cell scaffolds and are widely applied in regenerative medicine [1,2,16]. Their integration with stem cells or secretomes enables the design of bioactive nano-systems for effective wound repair [7,8,9]. Complementing these approaches, natural compounds such as luteolin (LUT) offer antimicrobial, anti-inflammatory, and antioxidant benefits. It exhibits potent antioxidant properties and inhibits inflammatory cascades, promoting anti-inflammatory macrophage polarization. It plays a crucial role in both diabetes management and wound healing following bacterial infections [17,18]. By eliminating bacteria in acidic pH environments and leveraging the high ROS characteristics of the inflammatory microenvironment to regulate drug release, it accelerates wound re-epithelialization, promotes angiogenesis, and facilitates tissue regeneration. When incorporated into ROS-responsive nanocarriers like poly (propylene sulfide)-poly (ethylene glycol) (PPS-PEG), smart drug delivery systems can be engineered for targeted therapeutic action. Furthermore, natural polymers including chitosan and alginate contribute biocompatibility, moisture retention, and immunomodulatory functions, forming ideal matrices for next-generation wound dressings [19,20,21].

Based on this rationale, we developed a chitosan/sodium alginate composite hydrogel loaded with luteolin nanoparticles and adipose-derived stem cells (denoted as GelCA@LUT@ADSCs). The hydrogel was fabricated via genipin-mediated cross-linking between cationic chitosan and anionic sodium alginate, with ADSCs encapsulated within its matrix. Luteolin was incorporated by means of ROS-responsive nanoparticles (LUT@NPs); these nanoparticles were prepared using PPS-PEG block copolymers and subsequently coated onto the hydrogel surface. This design enables spatiotemporal coordination: LUT@NPs initially modulate the inflammatory microenvironment, thereby facilitating the enhanced pro-healing functions of the encapsulated ADSCs. We systematically evaluated the physicochemical and biological properties of this composite system, with a particular focus on its dual regulatory roles in macrophage polarization and angiogenesis.

## 2. Material and Methods

### 2.1. Materials

Luteolin was purchased from TargetMol (Boston, MA, USA); chitosan (CS, 200–400 mPa·s), sodium alginate (SA, 200–500 mPa·s), genipin, DPPH and polyvinyl alcohol (PVA, alcoholysis degree 78.5–81.5 mol%; 2.8–3.3 mPa·s) were purchased from Aladdin (Shanghai, China); PPS-PEG (PPS polymerization degree 60, PEG molecular weight 2000) was purchased from Ruixi Biotechnology (Xi’an, China); trichloromethane and ethanol were purchased from Wansheng Chuandong (Chongqing, China). Fetal bovine serum and DMEM medium were purchased from Gibco (Waltham, MA, USA). Trypsin cell digestion solution and Triton X-100 was purchased from Beyotime Biotechnology (Shanghai, China). PBS was purchased from Dingguo Changsheng (Beijing, China). H_2_O_2_ was purchased from Northern Weiye Metrology Technology Research Institute (Beijing, China). DCFH-DA was purchased from Solarbio (Beijing, China). Triton X-100 was purchased from Beyotime Biotechnology (Shanghai, China). CCK-8 assay kit was obtained from MedChemExpress (Monmouth Junction, NJ, USA). Live/Dead Cell Staining Kit and Super Oxide Dismutase (SOD) Activity Assay Kit were acquired from Beibo Biotechnology (Shanghai, China). ELISA kits were supplied by AmyJet Scientific (Wuhan, China). *Staphylococcus aureus*, *Pseudomonas aeruginosa*, and cells were provided by the Department of Pharmaceutical Sciences, Laboratory of Special Formulation R&D, Army Medical Center, Chongqing, China. A total of 72 male Sprague Dawley (SD) rats weighing 180–220 g were purchased from Sichuan Viton Lever Experimental Animal Technology Co., Ltd. (Chengdu, China), with animal production license number: SCXK (Sichuan) 2023-0040.

### 2.2. Preparation of LUT@NPs

LUT@NPs were prepared via the single-emulsification solvent volatilization method. First, 2.14 mg of LUT was dissolved in 150 μL of ethanol, while 15 mg of PPS-PEG block copolymer was dissolved in 850 μL of chloroform. These two solutions were thoroughly mixed and then added dropwise into 6 mL of 0.5% (*w*/*v*) polyvinyl alcohol aqueous solution. The mixture was sonicated using a Q500 ultrasonic homogenizer (Qsonica, Newtown, CT, USA) at 4 °C with 50% amplitude for 10 min, followed by rotary evaporation at 33 °C for 10 min to remove organic solvents. Subsequently, the resulting dispersion was centrifuged at 2800× *g* for 5 min to eliminate large particle aggregates.

### 2.3. Preparation of GelCA@LUT@ADSCs

The preparation of the GelCA@LUT@ADSCs was conducted in two sequential stages: First, a 2% (*w*/*v*) CS solution was prepared by dissolving CS powder in a 2% (*v*/*v*) aqueous acetic acid solution under continuous stirring at room temperature until complete dissolution. Separately, a 2% (*w*/*v*) SA solution was prepared in deionized water. These two solutions were then mixed at three different volume ratios, V_CS_:V_SA_ = 1:1 (1 mL:1 mL), 1:2 (1 mL:2 mL), and 2:1 (2 mL:1 mL). Subsequently, genipin solution (0.5%, *w*/*v*) was added to the above CS-SA mixtures at four different dosages (0.5, 1.0, 1.5, and 2.0 mg, respectively). Mixed them under gentle magnetic stirring for 5 min, leading to the formation of a polyelectrolyte complex via electrostatic interaction. After thorough mixing, the mixtures were incubated at 37 °C for 24 h under light-protected conditions to induce cross-linking and form GelCA. Second, ADSCs were harvested and resuspended; the cell suspension was then added to the pre-prepared GelCA precursor after 2 h of incubation (prior to complete gelation). The ADSCs were washed with sterile PBS, and digested using 0.1% trypsin. After gentle trituration, the cell suspension was centrifuged. The supernatant was discarded, and the cell pellet was resuspended in a minimal volume of medium to obtain a concentrated ADSC suspension. For hydrogel fabrication, the GelCA precursor solution was first cross-linked. Subsequently, 2 × 10^6^ ADSCs were gently incorporated into the pre-formed GelCA hydrogel matrix to construct the GelCA@ADSCs composite. In parallel, a homogeneous LUT@NPs suspension was prepared. Homogenize the LUT@NPs suspension by briefly vortexing and sonicating in a water bath for 1 min prior to coating. The volume required for coating was calculated based on a predefined drug release profile. Following a 20-h incubation to ensure complete GelCA matrix formation. Using a micropipette, a precisely measured volume of LUT@NPs suspension was spirally dispensed onto the fully cured surface of the GelCA@ADSCs hydrogel. The coated hydrogel was then left to stand for 1 h to allow the film to adhere and dry, forming a continuous, uniform drug-loaded layer. This procedure yielded the final GelCA@LUT@ADSCs composite material.

### 2.4. Characterization of LUT@NPs

The morphology of LUT@NPs was observed using a transmission electron microscope (TECNAI 10, FEI, Hillsboro, OR, USA). The LUT@NPs were thoroughly mixed and diluted 500-fold with ddH_2_O. The diluted suspension was then drop-cast onto an electron microscopy copper grid pre-coated with a support film. After air-drying at room temperature, the sample was subjected to TEM observation. Particle size and polydispersion index (PDI) of LUT@NPs were examined using a laser particle sizer (ZS90, Malvern, Norcross, GA, USA) after a 5-fold dilution with ddH_2_O. Measurements were performed at a scattering angle of 90° and a constant temperature of 25 °C. High-performance liquid chromatography (HPLC) was utilized for drug loading rate (DLR), encapsulation efficiency (EE) and drug quantification. The EE and DLR were calculated as follows: $encapsulation\;efficiency\;(\%)={100\times m}_{1}/m_{2}$, and ${drug\;loading\;rate\;(\%)=100\times m}_{1}/\left(m_{1}+m_{PPS-PEG}\right)$, where $m_{1}$ is the actual drug loading as determined by HPLC, and $m_{2}$ is the theoretical dosage.

### 2.5. Characterization of GelCA@LUT@ADSCs

The morphology of GelCA@LUT@ADSCs was observed using a scanning electron microscope (S-3400N II, Hitachi, Tokyo, Japan). The chemical composition and structural characteristics of GelCA@LUT@ADSCs were determined by an FT-IR spectrometer (IRAffinity-1S, Shimadzu, Kyoto, Japan). The mechanical properties were tested by a rheometer (DHR-1, TA, Milford, MA, USA) under the following testing conditions: 37 °C, 10% deformation, and a frequency range of 1~20 Hz. To evaluate the water absorption capacity of the GelCA@LUT@ADSCs hydrogel, samples were immersed in an excess volume of PBS and the swelling ratio was determined at various time points. The swelling ratio was calculated using the following formula: $swelling\;ratio\;(\%)=(W_{t}-W_{d})/W_{d}\times 100\%\;$ [22]. W_t_ represents the weight of the swollen hydrogel at time “t”, and W_d_ denotes the initial weight of the dry hydrogel. Subsequently, key functional properties of GelCA@LUT@ADSCs were evaluated, including water content, water retention capacity, water vapor transmission rate, and drug release behavior. The detailed experimental procedure is described in the Supplementary Materials.

### 2.6. Anti-Inflammatory Activity

RAW264.7 cells were cultured in 6-well plates (density: 1 × 10^6^ cells/well) using DMEM with 10% fetal bovine serum at 37 °C, 5% CO_2_. A control group treated with 1000 ng/mL lipopolysaccharide (LPS, Solarbio, Beijing, China) (without the test material) was used to serve as the LPS-stimulated reference. The concentrations of inflammatory factors IL-6, IL-1β, and TNF-α in cell supernatants were detected by plotting standard curves with an ELISA kit.

### 2.7. Antioxidant Activity

For evaluating the protective effect of GelCA@LUT@ADSCs on cells under oxidative stress, RAW264.7 cells were first subjected to 800 μM H_2_O_2_ and serum-free co-culture for 8 h, then GelCA@LUT@ADSCs was added. Two control groups were set: ① H_2_O_2_-only control (no GelCA@LUT@ADSCs) and ② blank control (no H_2_O_2_, no GelCA@LUT@ADSCs). The antioxidant capacity of the GelCA@LUT@ADSCs was characterized by three key assays: DPPH assay (1,1-Diphenyl-2-picrylhydrazyl radical, for radical scavenging ability), DCFH-DA (2′,7′-Dichlorodihydrofluorescein diacetate) fluorescence staining (for intracellular ROS detection), and intracellular SOD activity measurement (for endogenous antioxidant enzyme evaluation). 100 mg of the material was immersed in 2 mL of DPPH solution (ethanol as solvent) at a concentration of 100 μM. After dark incubation for 1 h, the assay was carried out using a microplate reader at a wavelength of 517 nm.

The DPPH free radical scavenging activity was calculated as $free\;radical\;scavenging\;activity\;(\%)=100\times (A_{1}-A_{2})/A_{1}$, where A_1_ is the absorbance value of DPPH solution and A_2_ is the absorbance value of DPPH solution after incubation with different formulations. SOD activity was calculated as ${SOD\;activity\;(U/mL)=(A}_{3}-A_{4})/\left(50\%\times A_{3}\times V_{T}\right)$, where A_3_ and A_4_ are the absorbance values of the control and experimental groups, V_T_ is the volume (mL) used in the determination.

### 2.8. Cell Compatibility and Hemocompatibility

RAW264.7 cell line was provided by Zhongqiao Xinzhou Biotechnology Co., Ltd. (Shanghai, China), L929 cell line was obtained from Cell Bank, Type Culture Collection of Chinese Academy of Sciences (Shanghai, China), and HUVEC cell line was obtained from Dr. Xia Lei. RAW264.7, L929, and HUVEC cells were cultured in 24-well plates (density: 1 × 10^5^ cells/well) using DMEM with 10% fetal bovine serum at 37 °C, 5% CO_2_. After 12 h of incubation, GelCA@LUT@ADSCs were placed in cell-containing wells. The control group was left untreated. After 1, 2, and 3 days of culture, cell activity was detected using CCK-8 kit (MedChem Express, Monmouth Junction, NJ, USA). Then, cell survival was detected by live–dead staining after 3 days. Hemocompatibility of the GelCA@LUT@ADSCs was characterized by a direct contact hemolysis assay to evaluate its in vitro acute hemolytic capacity. For specific experimental methods, see the Supplementary Materials.

### 2.9. Antibacterial Activity

Gram-positive *Staphylococcus aureus* (*S. aureus*) and Gram-negative *Pseudomonas aeruginosa* (*P. aeruginosa*) were co-cultured with GelCA@LUT@ADSCs in 1 mL bacterial suspension (10^6^ CFU/mL) to determine the composite’s antimicrobial capacity. The bacterial suspension without any treatment was used as a control. They were incubated at 37 °C and 100 rpm for 24 h. The bacterial suspension was collected and 100 μL was taken after 10^3^-fold dilution and applied to tryptic soy agar. The colonies were counted after 24 h. Antimicrobial resistance was calculated as $antibacterial\;rate\;(\%)=100-100\times N_{1}/N_{2}$, where N_1_ is the number of bacterial colonies in the material-co-cultured group, and N_2_ is the number of colonies in the control group.

### 2.10. Animal Studies

All animal experiments in this study were approved and performed by the Laboratory Animal Welfare and Ethics Committee of the Army Medical University. All rats were anesthetized with sevoflurane (Sigma, St. Louis, MO, USA), shaved on the back, and sterilized with 75% alcohol before trauma was made. A 10 mm circular full skin defect trauma was made on the back of each rat. Afterward, the trauma was infected with 20.0 μL of *Pseudomonas aeruginosa* suspension (1 × 10^8^ CFU/mL), and modeling was considered successful after 6 h. Rats were divided into six groups (n = 6) and were given LUT@NPs, GelCA, GelCA@LUT, GelCA@ADSCs, GelCA@LUT@ADSCs and sterile PBS (control) treatments. GelCA@LUT@ADSCs (or other preparation) was used to treat the wounds every two days. A camera was used to capture the wound-healing process. Animals were sacrificed at the indicated times. Wound tissues were paraffin-embedded and sectioned for histological analysis. And the samples were stained with hematoxylin eosin (H&E) (Solarbio, Beijing, China), Masson trichrome stain (Servicebio, Wuhan, China), immunohistochemistry and immunofluorescence stain (AiFang biological, Changsha, China). The stained slides were imaged and analyzed. Quantitative analysis was performed using ImageJ 1.46.

### 2.11. Statistical Analysis

All quantitative data are expressed as mean ± standard deviation (SD). All experiments were conducted at least 3 times independently. Statistical differences were analyzed using one-way ANOVA and Student’s *t*-test. Statistical analysis was performed using GraphPad Prism 10.1.2 software. *p*-value < 0.05 was considered statistically significant.

## 3. Results and Discussion

### 3.1. Characterization of LUT@NPs and GelCA@LUT@ADSCs

**Figure 1:**
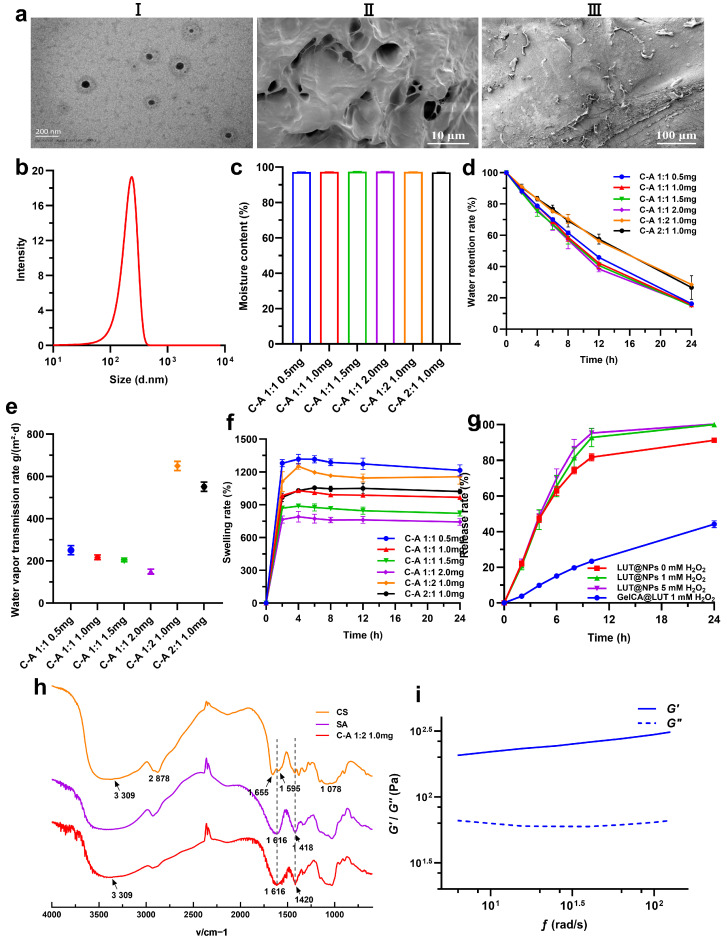
Characterization of GelCA@LUT@ADSCs hydrogel. (**aI**) Transmission electron microscopy image of LUT@NPs and (**aII**) scanning electron micrograph of GelCA@LUT@ADSCs showing a microporous structure after lyophilization, with a CS:SA ratio of 1:2 (1 mL:2 mL). (**aIII**) ADSCs successfully loaded into the hydrogel. (**b**) The particle size distribution, (**c**) water content, (**d**) water retention rate, and (**e**) water vapor transmission rate (WVTR), and (**f**) swelling ratio of GelCA@LUT@ADSCs with varying crosslinker concentrations and material mass ratios. (**g**) In vitro drug release profiles of LUT@NPs and GelCA@LUT@ADSCs. (**h**) Fourier transform infrared (FTIR) spectra of CS, SA, and GelCA@LUT@ADSCs. (**i**) Rheological properties of GelCA@LUT@ADSCs hydrogels. Data are presented as mean ± SD (n = 3).

We determined the optimal ratio of luteolin to PPS-PEG through comprehensive evaluation of particle size, PDI, EE, and DLR capacity, and the optimal ratio is 2.0:15.0 (luteolin:PPS-PEG). The particle size of LUT@NPs was 235 ± 5 nm with a PDI of 0.210 ± 0.013, an encapsulation efficiency (EE%) of 85.4 ± 1.0%, and a drug loading rate (DLR%) of 9.5 ± 0.1% (Figure 1(aI,b)). Strong electrostatic interactions between the cationic amino groups on the CS chain and the anionic carboxyl groups on the SA chain lead to the formation of a polyelectrolyte complex, namely a condensed layer. The amino acid reaction between genipin and CS builds a stable covalent cross-linked network on top of the aforementioned electrostatic composite network, thereby forming the final GelCA hydrogel. Scanning electron microscopy (SEM) analysis of GelCA@LUT@ADSCs revealed a densely porous internal architecture, indicative of good mechanical properties and stability (Figure 1(aII)). The successful incorporation of ADSCs was confirmed by their cellular adhesion and distribution patterns (Figure 1(aIII)). The morphological features of hydrogel pores, including density and spatial arrangement, fundamentally govern critical performance metrics, such as water vapor transmission rate, moisture retention capacity, mechanical strength, and swelling behavior. Water absorption rate, governed by material composition and crosslinking degree, critically influences hydrogel’s mechanical/tribological properties and modulates its drug release kinetics [23]. GelCA@LUT@ADSCs exhibited a moisture content exceeding 95% (Figure 1c), demonstrating exceptional moisture retention capacity. Although increased crosslinker concentration progressively densified the crosslinked network, water retention capability remained statistically indistinguishable among groups, with all samples decreasing below 16% after 24 h. Elevated material mass correlated with greater water retention rate, while SA demonstrated superior water retention properties compared to CA (Figure 1d). Water vapor transmission rate (WVTR) primarily depends on wound depth; deeper wounds result in greater moisture loss. A lower WVTR in dressings reduces water loss, while a higher WVTR increases it, which was measured at 204 g/(m^2^·d) for intact skin, with injured skin exhibiting a significantly higher value [24]. An optimal WVTR is critical for maintaining a moist wound interface, thereby fostering a microenvironment conducive to healing characterized by mild acidity, hypoxia, autolytic debridement, and growth factor release. This environment promotes granulation tissue formation and epithelial cell proliferation, ultimately accelerating wound closure [25]. In dry dressings, water vapor transmission rates are typically lower [26]. In acute wounds, exudate levels are higher during the initial 24–48 h compared to later stages. Dressings with WVTRs suited for early high exudate levels may dry out the wound later, while lower WVTRs suitable for later healing require removal or replacement to prevent fluid accumulation beneath the dressing [27]. Alginate dressings only need to cover the wound itself, and the appropriate replacement frequency should be determined. Experimentally, WVTR exhibited an inverse correlation with crosslinker concentration, decreasing from 250.7 ± 21.5 g/(m^2^·d) to 149.9 ± 11.1 g/(m^2^·d). This phenomenon is attributed to the enhanced crosslinking density within the hydrogel, which yields a more compact molecular network that impedes gas permeation. Notably, the GelCA@LUT@ADSCs formulation with a chitosan/sodium alginate (CS/SA) mass ratio of 1:2 demonstrated the highest WVTR of 650.2 ± 21.8 g/(m^2^·d) (Figure 1e). The low water vapor transmission rate (WVTR) of hydrogels ensures excellent moisture retention, keeping the wound moist during the later stages of healing and promoting optimal recovery.

Hydrogels exhibit rapid swelling in aqueous environments, retaining significant amounts of water without dissolution. This superior swelling capacity enables maintenance of a hydrated state, facilitating absorption of wound exudate and consequently reducing infection risks [28]. The swelling ratio of GelCA@LUT@ADSCs ranged from 716.4% to 1215.3%, exhibiting an inverse correlation with crosslinking density (Figure 1f). Subsequent contraction (negative swelling) was observed, attributable to the saturation of hydrophilic polymer segments and the consequent relaxation of the network structure, which exposed hydrophobic segments to the aqueous environment [29]. Ultimately, a dynamic equilibrium is established between the hydrophilic segments and hydrophobic domains of the polymer network. For the in vitro drug release profiles, LUT@NPs exhibited a significant ROS-responsive release behavior. Compared with the control group (0 mM H_2_O_2_), the release rate of LUT from LUT@NPs was significantly accelerated under the conditions of 1 mM and 5 mM H_2_O_2_. In contrast to the complete drug release of LUT@NPs within 24 h, GelCA@LUT exhibited a significantly attenuated release rate, with only 45.5% ± 6.6% of the payload released at the 24-h mark (Figure 1g). This release profile confirms the role of the hydrogel in facilitating sustained drug release. The incomplete release is likely attributable to the initial uniform coating of LUT@NPs on the GelCA surface, followed by their subsequent gradual infiltration into the hydrogel’s porous matrix. Based on the collective experimental evidence, the GelCA@LUT@ADSCs formulation with a CS/SA mass ratio of 1:2 was selected for subsequent investigation due to its optimal properties, which are particularly beneficial for managing chronic wounds characterized by high exudate. Comparative analysis of FTIR spectra for CS, SA, and GelCA@LUT@ADSCs revealed significant alterations in the hydrogel’s molecular structure. The broad band at 3309 cm^−1^ notably diminished in intensity. This is attributed to their interaction with the hydroxyl (OH) groups of genipin, which leads to the formation of covalent bonds between aldehyde and amine groups. Characteristic absorption peaks of CS at 1655 cm^−1^ and 1595 cm^−1^ merged into a broadened peak centered at 1616 cm^−1^ (Figure 1h). This spectral shift is attributed to the nucleophilic reaction between the primary amine groups (–NH_2_) of chitosan (CS) and the cross-linking agent, which results in the formation of covalent bonds [30], potentially supplemented by hydrogen bonding and electrostatic interactions between CS and SA. The distinctive peak at 1420 cm^−1^ corresponds to the ring stretching vibration of heterocyclic amines generated following the incorporation of genipin [31]. Furthermore, the hydrogel spectrum retained other characteristic peaks intrinsic to the individual CS and SA components. The mechanical properties of the hydrogel were substantially enhanced, owing to significant electrostatic interactions and hydrogen bonding between CS and SA, combined with covalent crosslinking. This synergistic integration of bonding mechanisms resulted in improved structural integrity. The formulation with a crosslinker concentration of 1.0 mg and a CS/SA mass ratio of 1:2 exhibited a storage modulus (G′) of 310 Pa (Figure 1i). The result indicate that the hydrogel is successfully prepared, with favorable moldability, good viscoelasticity, and a moderate hardness.

### 3.2. Cell Compatibility, Hemocompatibility and Antibacterial Ability

**Figure 2:**
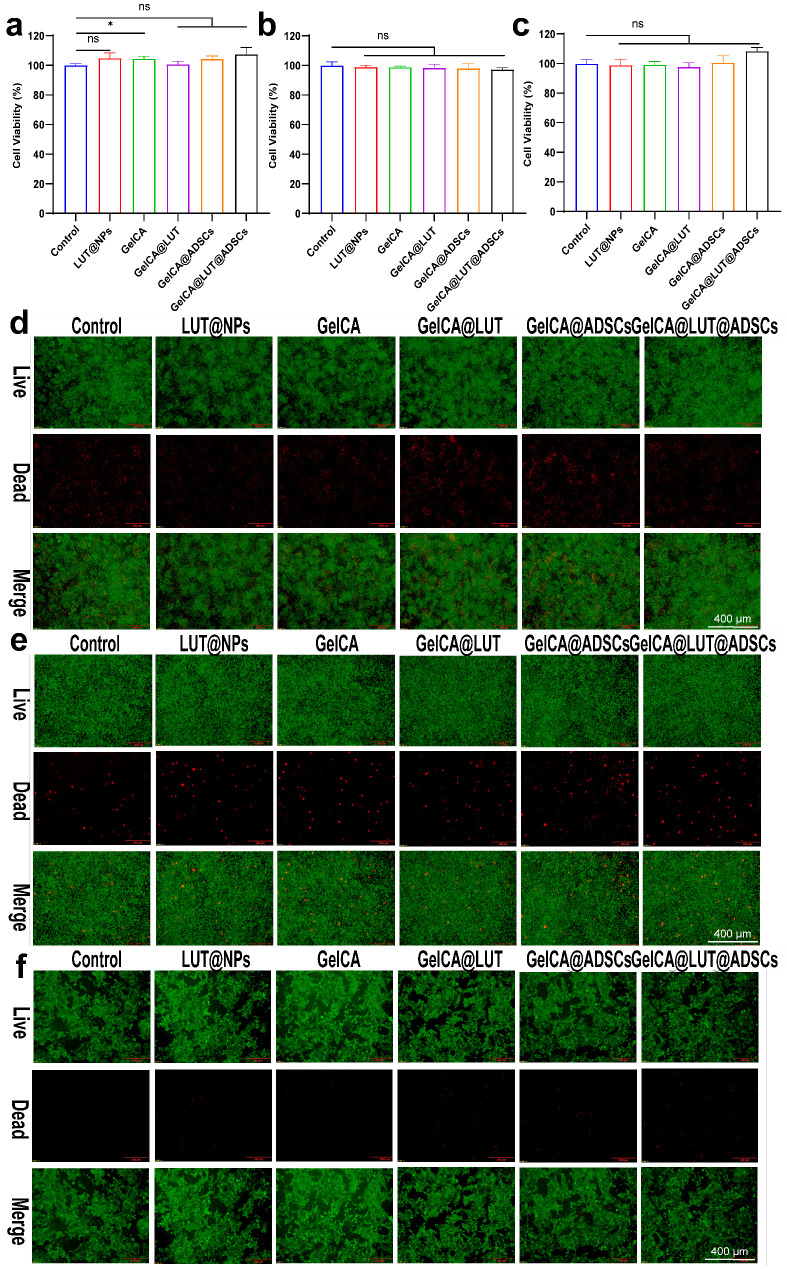
Cell viability of GelCA@LUT@ADSCs hydrogel. (**a**–**c**) CCK-8 assays and (**d**–**f**) live/dead cell staining after 72 h of co-culture with RAW264.7, L929, and HUVEC cells. The survival rate of cells co-cultured with GelCA@LUT@ADSCs for 72 h exceeded 95%, with live cells (green) and dead cells (red) at comparable levels to the control group. Data are presented as mean ± SD (n = 3). ns *p* > 0.05, * *p* < 0.05, compared with the control group.

**Figure 3:**
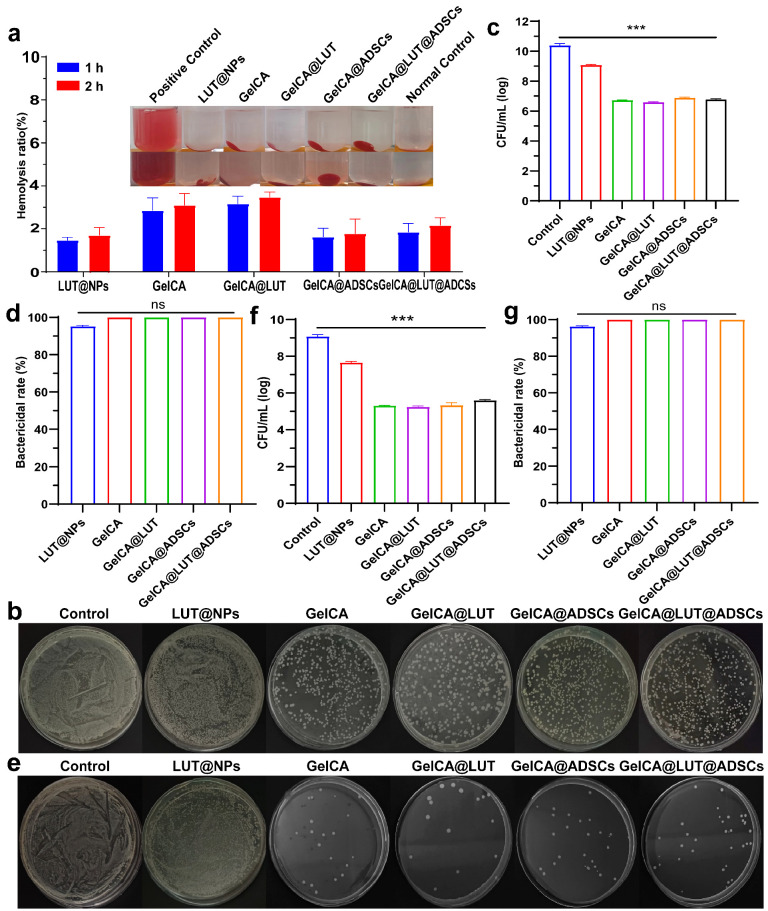
Hemolysis assay and antibacterial activity of GelCA@LUT@ADSCs hydrogel. (**a**) Hemolysis assay of GelCA@LUT@ADSCs. No significant hemolysis was observed, with a hemolysis rate below 5% after 2 h, indicating that GelCA@LUT@ADSCs exhibit excellent blood compatibility. Antibacterial activity of GelCA@LUT@ADSCs against *S. aureus*: (**b**,**c**) bacterial colony counts and (**d**) inhibition rate. Antibacterial activity against *P. aeruginosa*: (**e**,**f**) bacterial colony counts and (**g**) inhibition rate. GelCA@LUT@ADSCs demonstrated potent antibacterial activity against both *Staphylococcus aureus* and *Pseudomonas aeruginosa*, with inhibition rates exceeding 99% for both strains. Data are presented as mean ± SD (n = 3). ns *p* > 0.05, *** *p* < 0.001, compared with the control group.

Cytocompatibility is the basis of wound dressing. As a medical material that directly contacts wound tissue, the ability of a wound dressing to establish “friendly” interactions with the local cells of the wound directly determines the application value of the dressing and the final effect of wound healing. RAW264.7 macrophages, L929, and HUVEC cells were co-cultured with LUT@NPs, GelCA, GelCA@LUT, GelCA@ADSCs, and GelCA@LUT@ADSCs for 1, 2, and 3 days, respectively. Cell survival was observed. We could observe that cell viability of GelCA@LUT@ADSCs exceeded 95%, demonstrating excellent cytocompatibility (Figure 2a–c and Figure S1a–f). Live/dead staining visualized that GelCA@LUT@ADSCs-treated cells were at the same level as the control (green and red indicate live and dead cells, respectively) (Figure 2d–f). No significant erythrocyte lysis was observed, with hemolysis rates remaining below 5% at both 1 h and 2 h, indicating good hemocompatibility of GelCA@LUT@ADSCs (Figure 3a). The slightly higher hemolysis rates in two groups (GelCA and GelCa@LUT) may be attributed to subtle variations in the experimental operation process. Specifically, the mixing intensity or incubation time of the sample with red blood cell suspension might have deviated slightly from the standard protocol, leading to a marginal increase in hemolysis. Notably, all values (including the two higher groups) are well below the hemolysis standard (5%), indicating acceptable biocompatibility. Bacterial infection exacerbates wound exudate production, induces immune system inflammatory responses, and creates an elevated ROS (reactive oxygen species) microenvironment, all of which impede wound healing. Wound dressings with antimicrobial capabilities can effectively prevent such infections, particularly the bacterial infections associated with impaired wound healing. GelCA@LUT@ADSCs exhibited potent antibacterial activity against both *S. aureus* and *P. aeruginosa*, with inhibition rates of 99.98 ± 0.001% and 99.97 ± 0.003%, respectively (Figure 3b–g).

It is noteworthy that the composition of wound exudate (proteins, electrolytes, enzymes, etc.) significantly influences drug diffusion and bacterial behavior. To avoid damaging the surrounding skin, advanced dressings must possess the ability to drive exudate absorption toward the outer surface (vertical diffusion) [32,33]. However, our study did not incorporate microbiological testing under conditions involving wound exudate, presenting certain limitations.

### 3.3. In Vitro Anti-Inflammatory and Antioxidant Properties

GelCA@LUT@ADSCs exhibits significant anti-inflammatory effects by suppressing the production of inflammatory mediators, reducing inflammatory responses, and protecting cells from oxidative stress damage.

**Figure 4:**
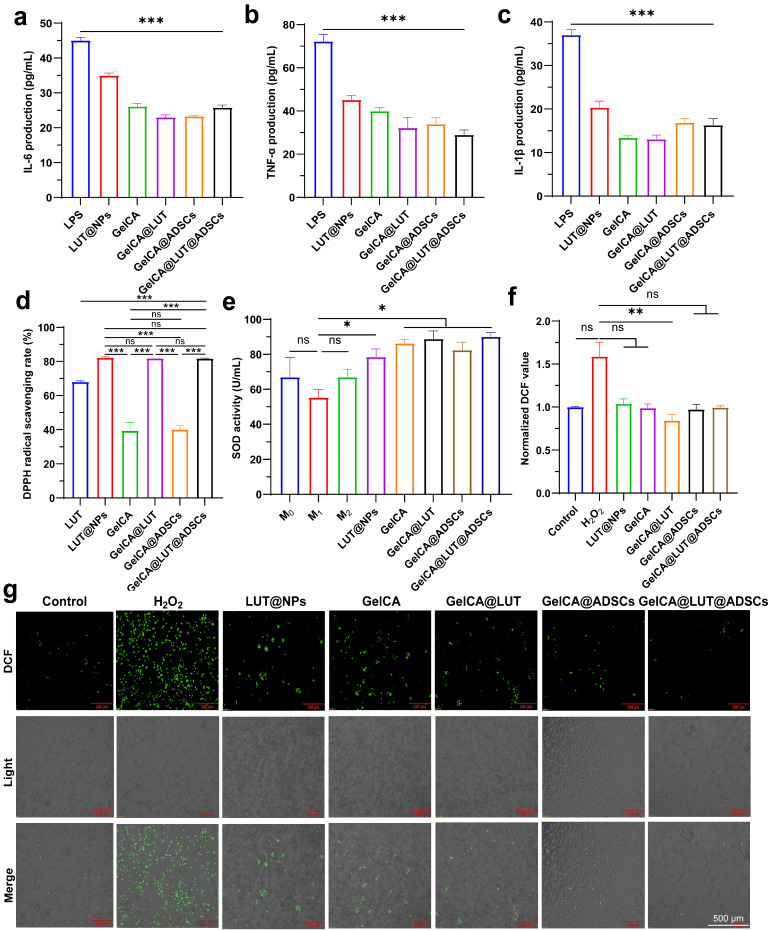
In vitro anti-inflammatory and antioxidant capacity of GelCA@LUT@ADSCs hydrogel. (**a**–**c**) Expression levels of (**a**) IL-6, (**b**) TNF-α, and (**c**) IL-1β in RAW 264.7 cells after 24 h of co-culture with GelCA@LUT@ADSCs. (**d**) DPPH radical scavenging rate of GelCA@LUT@ADSCs. (**e**) SOD enzyme activity. (**f**,**g**) DCF fluorescent probe-based observation and quantitative analysis in RAW 264.7 cells after 24 h of co-culture with GelCA@LUT@ADSCs. Green indicates ROS intensity. Data are presented as mean ± SD (n = 3). ns *p* > 0.05, * *p* < 0.05, ** *p* < 0.01, *** *p* < 0.001, compared with LPS/H_2_O_2_ group.

To evaluate the anti-inflammatory efficacy in vitro, the expression levels of inflammatory cytokines IL-6, TNF-α, and IL-1β were quantified using ELISA in RAW 264.7 cells following 24 h of LPS stimulation and co-culture with LUT@NPs, GelCA, GelCA@LUT, GelCA@ADSCs, and GelCA@LUT@ADSCs. In the LPS-induced inflammation model group (positive control), the levels of IL-6, TNF-α, and IL-1β were 45.1 ± 0.7 pg/mL, 72.3 ± 2.8 pg/mL, and 37.0 ± 1.1 pg/mL, respectively. Compared to the positive control group, all treatment groups showed a significant reduction in the release of these inflammatory cytokines (Figure 4a–c). These results suggest that GelCA@LUT@ADSCs mitigates the inflammatory response by decreasing the secretion of pro-inflammatory factors.

Both the base material and LUT can effectively mitigate free radical-induced cellular damage, underscoring their synergistic antioxidant capacity. In the DPPH assay, free luteolin was used as a control group to compare the antioxidant activity. The results showed that compared with free luteolin, both the LUT@NPs group and the nanoparticle-containing hydrogel groups exhibited higher DPPH radical scavenging capacity (~80%), indicating their superior antioxidant activity (Figure 4d). Subsequently, we investigated whether the hydrogel could enhance macrophage antioxidant capacity through a two-pronged approach: scavenging exogenous ROS and stimulating endogenous antioxidant defenses. Superoxide dismutase (SOD), a key antioxidant enzyme for protecting cells from oxidative stress damage, scavenges excess free radicals, suppresses inflammation, and maintains intracellular redox homeostasis, acting as a critical component of the endogenous antioxidant defense system. All treatment groups exhibited significantly higher SOD activity compared to the M_1_ model group (55.4 ± 3.6 U/mL). Notably, the SOD activity in the GelCA@LUT@ADSCs group reached 92.2 ± 1.8 U/mL, surpassing that of the M_2_ model group (67.0 ± 3.6 U/mL), indicating its potent ability to enhance cellular antioxidant capacity (Figure 4e). Following the validation of its endogenous antioxidant capacity, we employed DCFH-DA immunofluorescence staining to assess whether it could enhance macrophage antioxidant defense by scavenging exogenous ROS. Reduced DCF fluorescence intensity indicated that GelCA@LUT@ADSCs effectively alleviated ROS-induced damage under 24 h of H_2_O_2_-stimulated oxidative stress (Figure 4f,g). Collectively, GelCA@LUT@ADSCs enhances macrophage antioxidant capacity through a dual mechanism: clearing exogenous ROS and stimulating endogenous antioxidant defenses.

### 3.4. Wound Healing Analysis

#### 3.4.1. Wound Closure Analysis

**Figure 5:**
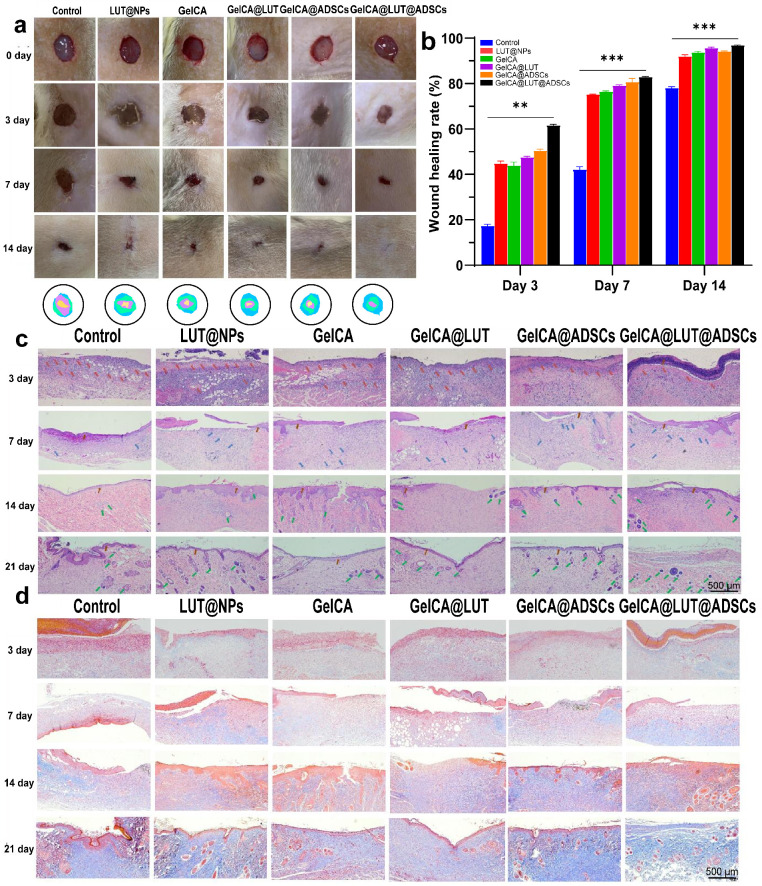
In vivo assessment of the GelCA@LUT@ADSCs for wound healing (n = 6). (**a**,**b**) Wound healing observation and quantification of bacterially colonized skin defects treated with different formulations. Blue, green, purple, and yellow represent wound size on days 0, 3, 7, and 14, respectively. (**c**) Representative H&E staining results of wound tissues. Red arrows indicate inflammatory cells, blue arrows denote newly formed capillaries, brown arrows highlight the newly regenerated epidermis, and green arrows point to nascent sebaceous gland structures. (**d**) Representative images of Masson’s trichrome staining. Muscle fibers and cytoplasm are stained red, while collagen fibers appear blue. Data are presented as mean ± SD (n = 6). ** *p* < 0.01, *** *p* < 0.001, compared with the control group.

A full-thickness wound model with bacterial infection was established in Sprague Dawley (SD) rats to evaluate the efficacy of GelCA@LUT@ADSCs as a wound dressing in promoting wound healing. Wound healing progression was monitored by capturing wound images at predetermined time points, followed by quantitative analysis of wound healing rate. Representative photographs of bacteria-infected wounds treated with different formulations are shown in Figure 5a. Macroscopic evaluation indicated that although all wounds gradually healed over the experimental period, significant variations in healing outcomes were observed among the treatment groups. For the control group, the wound area remained substantially larger on day 3, and residual scabbing was still evident even at day 14. In contrast, all treatment groups exhibited varying degrees of wound contraction, which was more pronounced than that observed in the control group. By day 7, extensive re-epithelialization was observed with minimal scarring. At day 14, wounds in the GelCA@LUT@ADSC group achieved nearly complete closure, whereas wounds in the control and other treatment groups remained partially open. Quantitative analysis of wound healing rate confirmed these observations (Figure 5b), showing that the GelCA@LUT@ADSC group achieved a superior wound healing rate of 96.93%, whereas the wound area closure in the control group was only 78.00%. These results indicate that GelCA@LUT@ADSCs effectively promote the repair of bacteria-infected wounds by inhibiting bacterial growth, alleviating inflammatory responses, stimulating the proliferation of tissue cells, and ultimately accelerating wound contraction. In addition, the desirable functionality of the GelCA@LUT@ADSCs and the moist environment provided are also beneficial to the wound healing.

The wound surface was covered by a visible eschar, beneath which epidermal regeneration, granulation tissue proliferation, neovascularization, and inflammatory cell infiltration were observed. In the control group, extracellular matrix (ECM) deposition was irregular, with sparse ECM distribution in localized areas. In contrast, the GelCA@LUT@ADSC group exhibited more abundant, uniformly dense ECM deposition along with evident re-epithelialization. By day 14, the granulation tissue appeared more mature than other groups, with newly formed sebaceous glands visible in the nascent dermal layer. At day 21, the wound was fully healed, showing well-organized glandular structures and surrounding tissue, with even distribution of sebaceous glands and hair follicles (Figure 5c). Furthermore, the GelCA@LUT@ADSC group exhibited readily detectable collagen deposition, active granulation tissue proliferation, and early neodermis formation by day 7 (Figure 5d). Collagen, the primary structural protein in the human body, plays a critical role in the wound healing process. Abnormal deposition or degradation of collagen during healing can impede tissue repair and lead to fibrosis. Type III collagen is a major component of the dermal ECM, accounting for approximately 10% of dermal collagen. It combines with Type I and Type V collagen to form organized collagen fibril bundles. These results collectively indicate that GelCA@LUT@ADSCs promotes cell recruitment, facilitates ECM deposition, stimulates structured collagen formation, and ultimately accelerates wound healing.

#### 3.4.2. Angiogenesis and Anti-Inflammatory In Vivo

**Figure 6:**
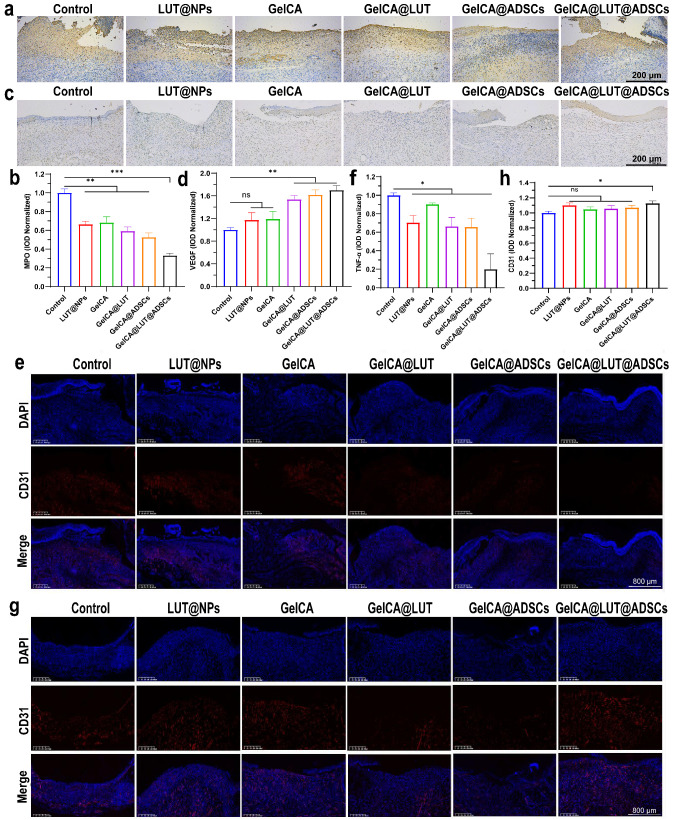
Histochemical analysis and related factor expression levels in wound tissues treated with different formulations. (**a**–**d**) Representative immunohistochemical (IHC) images and ImageJ-based quantification of MPO expression on day 3 and VEGF expression on day 7. Brown indicates MPO/VEGF. (**e**–**h**) Representative immunofluorescence (IF) images and ImageJ-based quantification of TNF-α expression on day 3 and CD31 expression on day 7. TNF-α and CD31 were labeled with red fluorescence, while cell nuclei were counterstained with blue fluorescence (DAPI). Data are presented as mean ± SD (n = 6). ns *p* > 0.05, * *p* < 0.05, ** *p* < 0.01, *** *p* < 0.001, compared with the control group.

Myeloperoxidase (MPO) serves as a marker for neutrophil activation. Its levels and activity reflect the functional status of granulocytes and are involved in regulating multiple processes of the inflammatory response [34,35]. Vascular endothelial growth factor (VEGF) is a specific pro-vascular endothelial growth factor that promotes increased vascular permeability, ECM degeneration, vascular endothelial cell migration, proliferation, and angiogenesis. Neovascularization is essential to transport nutrients and oxygen to the wound to maintain fibroblast proliferation, collagen synthesis, and re-epithelialization [36,37]. MPO and VEGF were localized and characterized via immunohistochemistry in wound tissues on post-wounding days 3 and 7, respectively. The experimental group showed a significant decrease in MPO and an increase in VEGF compared to the control group (brown). Obvious factor aggregation was observed at the site of re-epithelialization in the GelCA@LUT@ADSC group (Figure 6a–d). It accelerates wound healing by inhibiting neutrophil infiltration and pro-inflammatory cytokine production during the inflammatory phase and promoting angiogenesis in damaged skin.

**Figure 7:**
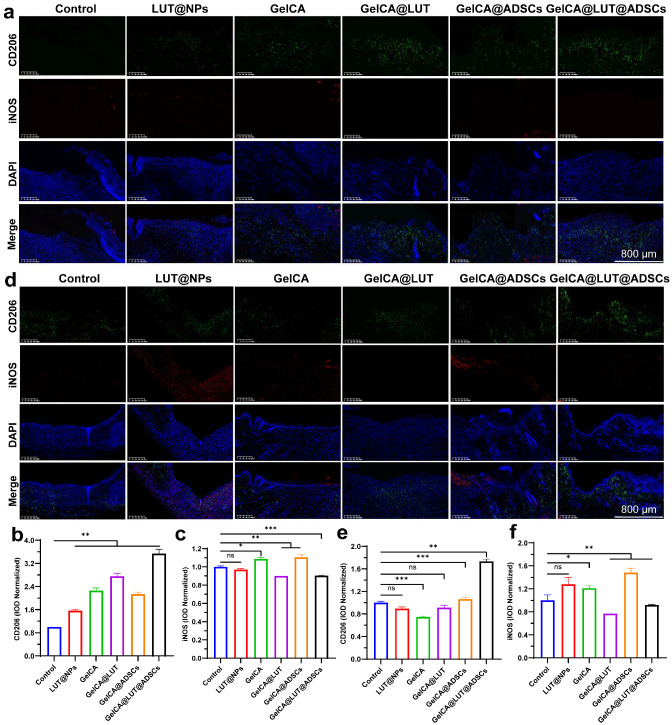
Expression levels of relevant factors in wound tissue treated with different formulations. (**a**–**c**) Representative IF images and quantitative analysis of M_1_ macrophage marker iNOS (inducible nitric oxide synthase) and M_2_ macrophage marker CD206 on day 3. (**d**–**f**) Representative IF images and quantitative analysis of iNOS and CD206 on day 7. iNOS is shown in red fluorescence, CD206 in green fluorescence, and cell nuclei in blue fluorescence (DAPI). Data are presented as mean ± SD (n = 6). ns *p* > 0.05, * *p* < 0.05, ** *p* < 0.01, *** *p* < 0.001, compared with the control group.

**Figure 8:**
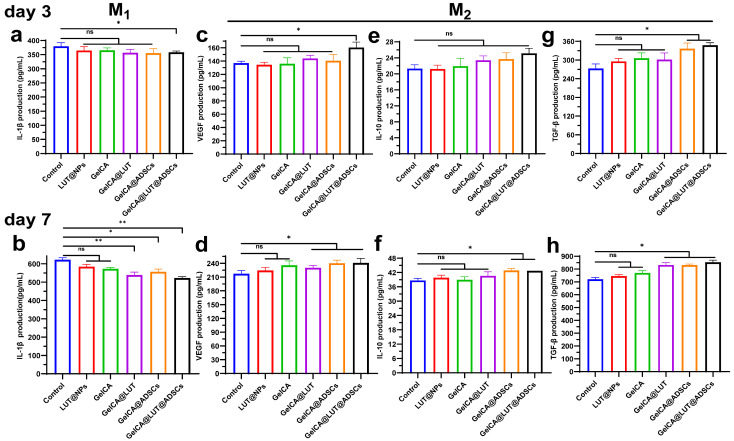
Expression of relevant factors in wound tissue during the inflammatory and proliferative phases. Quantitative analysis of (**a**,**b**) pro-inflammatory cytokine IL-1β, (**c**,**d**) anti-inflammatory cytokine VEGF, (**e**,**f**) pro-angiogenic factor IL-10, and (**g**,**h**) TGF-β in wound tissues on days 3 and 7. Data are presented as mean ± SD (n = 3). ns *p* > 0.05, * *p* < 0.05, ** *p* < 0.01, compared with the control group.

Chronic wounds are characterized by excessive inflammation, heightened protein hydrolysis, and impaired matrix deposition. Tumor necrosis factor-α (TNF-α), a key pro-inflammatory cytokine, plays a critical role in the inflammatory phase of wound healing [38]. Specifically, it promotes the recruitment of immune cells, activates nuclear factor NF-κB, and disrupts the balance between M_1_ and M_2_ macrophages—ultimately triggering persistent inflammation that undermines subsequent stages of wound healing [39]. Platelet endothelial cell adhesion molecule-1 (PECAM-1/CD31) is expressed in platelets, neutrophils, monocytes, and various immune cells; it participates in processes such as leukocyte migration and angiogenesis, and also plays an important role in the clearance of senescent neutrophils in vivo [40,41]. Therefore, we detected and quantified TNF-α and CD31 via immunofluorescence (IF) staining in tissues at 3 and 7 days following gel treatment. In this way, we evaluated the effects of GelCA@LUT@ADSCs, including the inhibition of neutrophil infiltration during the inflammatory phase, the suppression of pro-inflammatory cytokine production, and the promotion of neoangiogenesis in the proliferative phase. Compared with the other groups, the GelCA@LUT@ADSC group exhibited a significant reduction in the TNF-α level, enhanced matrix synthesis, and a marked increase in CD31 expression, indicating it had the optimal therapeutic effect (Figure 6e–h). In addition, its M_2_ macrophage marker CD206 (green) was increased considerably and M_1_ macrophage marker iNOS (red) was reduced compared to the control group, showing that it could promote the conversion of macrophages to the anti-inflammatory/pro-healing M_2_ phenotype and promote wound healing (Figure 7a–f). IL-1β levels were significantly downregulated in tissues following GelCA@LUT@ADSCs treatment, with an approximate 100 pg/mL difference observed on day 7 (Figure 8a,b). In contrast, the vascular endothelial growth factor (VEGF), as well as the anti-inflammatory factor IL-10 and pro-angiogenic factors TGF-β1, were upregulated to varying degrees, all of which showed statistically significant differences compared with the control group (Figure 8c–h). These suggest that GelCA@LUT@ADSCs could promote macrophage conversion to the anti-inflammatory/pro-healing M_2_ phenotype and accelerate wound healing by facilitating angiogenesis in damaged skin.

Luteolin is a flavonoid natural compound exhibiting multiple pharmacological activities such as antibacterial and anti-inflammatory effects. However, its clinical application is limited due to issues like poor water solubility and low bioavailability. Encapsulating luteolin within carriers such as nanoparticles, liposomes, or solid lipid nanoparticles can effectively achieve sustained-release drug delivery, thereby improving solubility, enhancing bioavailability, and increasing safety.

Currently, natural polymer hydrogels based on polysaccharides, such as alginates, chitosan, and cellulose, have been extensively studied due to their advantages of being renewable, biodegradable, and exhibiting excellent biocompatibility. Both chitosan and sodium alginate are natural polysaccharides that can form three-dimensional network structures through physical crosslinking (e.g., sodium alginate with calcium ions) or chemical crosslinking via covalent interactions [42]. Although SA readily forms gels through crosslinking with multivalent cations, its mechanical properties are typically poor. Combining it with other polymers (e.g., chitosan) can enhance mechanical performance while serving as a drug carrier for controlled drug release [43].

For cross-linking agent selection, the core advantages of selecting genipin are manifold: first, it boasts high biosafety as it is derived from the hydrolysis product of natural geniposide, exhibiting no cytotoxicity while its degradation products are free of toxic or side effects; second, it undergoes a mild crosslinking reaction that requires no harsh conditions and can be completed under physiological environments (e.g., normal temperature, neutral pH), thus effectively protecting bioactive components; third, the crosslinked products it forms feature stable performance—the hydrogel generated has moderate mechanical strength, a controllable swelling degree, and superior degradation resistance in comparison with many chemical crosslinking agents; fourth, it demonstrates strong reaction specificity, primarily reacting with amino groups and thereby causing minimal structural damage to biomacromolecules such as proteins and polysaccharides [44,45].

## 4. Conclusions

In this study, we developed an inflammation-microenvironment-responsive drug delivery system by fabricating luteolin-loaded nanoparticles (LUT@NPs) using PPS-PEG as the carrier. These nanoparticles were then coated onto a chitosan/alginate (GelCA) hydrogel matrix preloaded with adipose-derived stem cells (ADSCs), yielding the composite wound dressing GelCA@LUT@ADSCs. The physicochemical properties and biological functions of the system were systematically evaluated to elucidate its mechanisms in promoting wound healing. GelCA@LUT@ADSCs exhibited excellent biocompatibility and facilitated macrophage polarization toward the pro-healing M_2_ phenotype. It significantly accelerated wound healing through multiple mechanisms, including reduction in inflammation and hemorrhage, promotion of cell proliferation and migration, ECM remodeling, re-epithelialization, angiogenesis, hair follicle regeneration, and collagen deposition. Specifically, it downregulated pro-inflammatory factors (IL-1β, TNF-α) and MPO, while upregulating anti-inflammatory and pro-angiogenic mediators such as IL-10, TGF-β1, VEGF, and CD31. Overall, GelCA@LUT@ADSCs provides a protective moist wound environment, enhances angiogenesis and tissue regeneration, and significantly promotes healing through synergistic antibacterial, anti-inflammatory, and antioxidant effects. These findings underscore the promising potential of GelCA@LUT@ADSCs as an advanced therapeutic strategy for wound repair and regenerative medicine.

## Data Availability

The datasets generated and/or analyzed during the current study are available from the corresponding authors upon reasonable request.

## Supplementary Materials

The following supporting information can be downloaded at: https://www.mdpi.com/article/10.3390/pharmaceutics18010098/s1, Figure S1: Cytocompatibility of GelCA@LUT@ADSCs (n = 3). CCK-8 assay of (a,b) RAW264.7, (c,d) L929, and (e,f) HUVEC cells after 24 h and 48 h of co-culture. Cell viability exceeded 95%, demonstrating excellent cytocompatibility. Other methodological details conducive to experimental reproducibility include: Determination of Hydrogel Water Content, Water Retention Rate Measurement, WVTR, In Vitro Drug Release Behavior, and Hemolysis Test.
