# Supplementary material for: A Multifunctional Hydrogel Incorporating Luteolin-Encapsulated ROS-Responsive Nanoparticles and Stem Cells Promotes Bacterial-Infected Wound Healing
Source: Pharmaceutics. 2026 Jan 12;18(1):98. doi: 10.3390/pharmaceutics18010098 (PMC12845210; doi:10.3390/pharmaceutics18010098)
Supplement: Supplementary file 1 [file pharmaceutics-18-00098-s001.zip › pharmaceutics-3969124-supplementary.pdf]

Supplementary Methods

1. Determination of Hydrogel Water Content

The water content of GelCA@LUT@ADSCs was determined by a gravimetric method. Briefly, the as-prepared hydrogel was immediately weighed to obtain the initial mass (W_e) and then dried to a constant weight (W_d). The water content was calculated using the following formula: Water Content (%) = $[(W_e - W_d) / W_e] \times 100\%$. All measurements were performed in triplicate (n=3).

2. Water Retention Rate Measurement

The water retention rate of the hydrogels was evaluated under simulated wound conditions. The as-prepared hydrogels (W_e) were placed in an incubator maintained at 37°C. The weight of the hydrogels (W_t) was recorded at predetermined time intervals. The water retention ratio at time *t* was calculated as follows: Water Retention (%) = $(W_t / W_e) \times 100\%$. The experiment was continued until the hydrogel weight stabilized. Data are presented as mean ± SD (n=3).

3. Water Vapor Transmission Rate (WVTR)

The water vapor transmission rate (WVTR) was determined using a modified gravimetric cup method. Specifically, a cylindrical container (1.5 cm in diameter) filled with 9 mL of anhydrous calcium chloride was sealed with the tested hydrogel, which served as the only vapor-permeable barrier. The initial total weight (W_0) of the assembly was recorded. The setup was then placed in a desiccator maintained at 37 °C and 90% relative humidity. After 24 hours, the weight (W_t) was measured. The WVTR was calculated using the following formula: WVTR ($g \cdot m^{-2} \cdot day^{-1}$) = $(W_0 - W_t)$

/A, where A represents the exposed area of the hydrogel (in m^2). Results were obtained from three independent replicates (n = 3).

4. In Vitro Drug Release Behavior

The release profile of Luteolin (LUT) from the composite hydrogels was investigated using a dialysis method in PBS-1 mM (or 5 mM) H_2O_2 (pH 7.4, contains 1% (v/v) Tween-80) at 37° C. Briefly, pre-swollen hydrogel samples containing a known amount of LUT were placed in dialysis bags (MWCO: 3.5 kDa). The bags were then immersed in release medium under gentle shaking (100 rpm). At predetermined time points, 1 mL of the external release medium was withdrawn and replaced with an equal volume of fresh pre-warmed PBS to maintain sink conditions. The concentration of released LUT was quantified using high-performance liquid chromatography (HPLC) with a C18 column and a UV detector set at 349 nm. The chromatographic column model is Dikma Inspire C18 (150 mm × 4.6 mm, 5 μm). The detection wavelength is 349 nm, with a column temperature of 35°C. The eluent consists of methanol-water (0.1% phosphoric acid), volume ratio is 55:45, and the flow rate is 1.0 mL/min. All release studies were conducted in triplicate (n=3).

5. Hemolysis Test

Collect fresh rabbit blood using EDTA-K2 anticoagulant tubes. Centrifuge at 2000 rpm/min for 10 minutes. Aspirate the supernatant. Add physiological saline to wash the red blood cells, repeating this step three times. Resuspend the resulting red blood cell pellet in physiological saline to prepare a 2% (v/v) red blood cell suspension for later use. Add 1 mL each of the red blood cell suspension and extraction solution in a 1:1 ratio. For the negative control, replace the extraction solution with 1 mL physiological saline. For the positive control, replace the extraction solution with a 1%

Triton X-100 physiological saline solution. Incubate at 37°C for 1-2 hours, centrifuge at 2000 rpm for 5 minutes, collect the supernatant, and measure the optical density (OD) at 545 nm using a microplate reader.

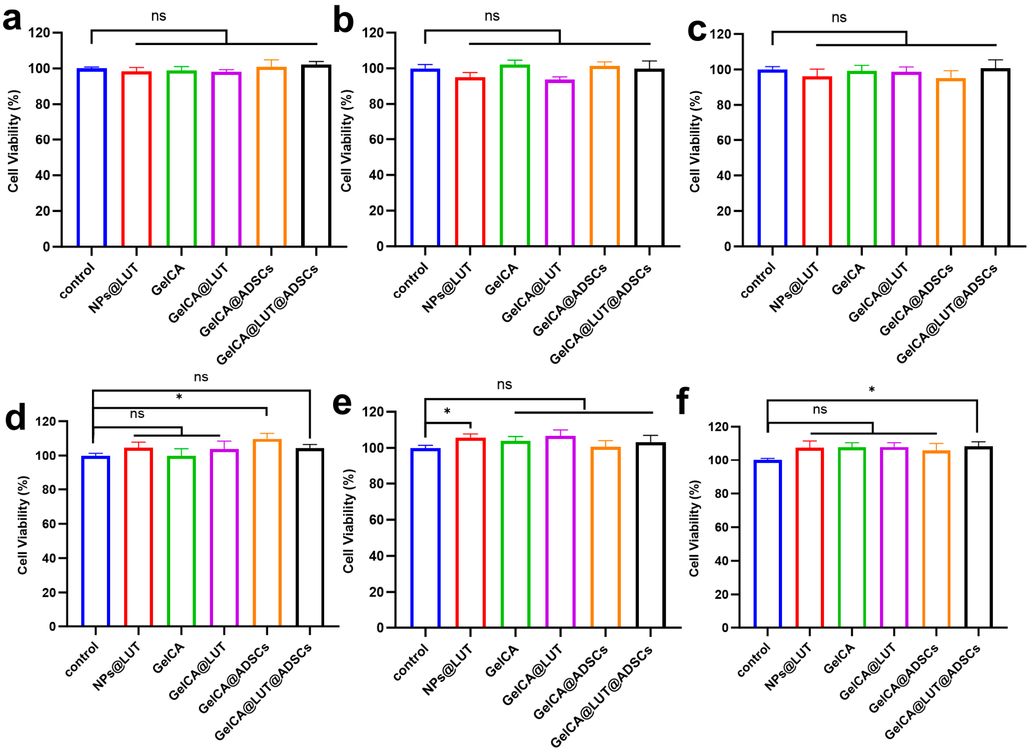

Figure S1. Cytocompatibility of GelCA@LUT@ADSCs (n=3). CCK-8 assay of (a-b) RAW264.7, (c-d) L929, and (e-f) HUVEC cells after 24 h and 48 h of co-culture. Cell viability exceeded 95%, demonstrating excellent cytocompatibility.
